# Is birth weight associated with pregestational maternal BMI? BRISA Cohort, Ribeirão Preto, Brazil

**DOI:** 10.1590/1414-431X202010037

**Published:** 2020-12-07

**Authors:** K.S.D. Trombe, L.S. Rodrigues, L.M.P. Nascente, V.M.F. Simões, R.F.L. Batista, R.C. Cavalli, C. Grandi, V.C. Cardoso

**Affiliations:** 1 Programa de Pós-Graduação em Saúde da Criança e do Adolescente, Faculdade de Medicina de Ribeirão Preto, Universidade de São Paulo, Ribeirão Preto, SP, Brasil; 2 Departamento de Puericultura e Pediatria, Faculdade de Medicina de Ribeirão Preto, Universidade de São Paulo, Ribeirão Preto, SP, Brasil; 3 Departamento de Saúde Pública, Universidade Federal do Maranhão, São Luís, MA, Brasil; 4 Departamento de Ginecologia e Obstetrícia, Faculdade de Medicina de Ribeirão Preto, Universidade de São Paulo, Ribeirão Preto, SP, Brasil; 5 Research Committee, Argentine Society of Pediatrics, Buenos Aires, Argentina

**Keywords:** Birth weight, Body mass index, Cohort study, Overweight, Obesity

## Abstract

Given the increase of women with excess weight or obesity and its possible effects on birth weight, the present study aimed to investigate the association between pregestational maternal body mass index (BMI) and birth weight in a birth cohort from Ribeirão Preto, SP, Brazil. This was a prospective study conducted on 1362 mother-child pairs involving singleton births. The women were evaluated using standardized questionnaires during the second trimester of pregnancy and at the time of childbirth. Information about the newborns was obtained from their medical records. The dependent variable was birth weight, categorized as low, adequate, or high. The independent variable was pregestational maternal BMI, categorized as malnutrition, adequate weight, overweight, and obesity. A multinomial regression model was used to estimate the crude and adjusted relative risk (RR) of low and high birth weight. A high frequency of pregestational excess weight (39.6%) was detected and found to be independently associated with high birth weight (RR=2.13, 95%CI: 1.19-3.80 for overweight and RR=3.34, 95%CI: 1.80-6.19 for obese pregnant women). There was no association between pregestational malnutrition and low birth weight (RR=1.70; 95%CI: 0.81-3.55). The present data showed a high rate of women with excess pregestational weight, supporting the hypothesis that pregestational BMI may contribute to high birth weight babies and indicating the need for actions aiming to prevent excessive weight in women at reproductive age.

## Introduction

Birth weight is considered the main indicator of newborn health in both epidemiological studies and clinical practice given its strong association with morbidity-mortality at the beginning of life (1 [Bibr B02]). Low- and high-birth weight newborns (NB) have a higher risk of perinatal death and other negative outcomes during childhood, adolescence, and adulthood (1–3).

Birth weight is the result of the interaction of biological, socioeconomic, and psychological factors. Among the biological factors, particularly important are the genetic background of mother and fetus, the maternal nutritional and metabolic status, the exposure of the binomial to diseases and toxins, the functioning of the placenta, and finally, the obstetric characteristics (4). Several authors have also considered factors such as pregestational maternal weight and height and maternal weight gain during pregnancy to be strongly associated with birth weight (5–7).

Studies have shown that pregestational maternal malnutrition may increase the risk of preterm birth, low birth weight, and small for gestational age (SGA) NB (8–10). Conversely, excess pregestational weight increases the risk of high birth weight and large for gestational age (LGA) NB (9,11,12), which in turn is related to overweight and/or obesity during the life cycle (9,11). Also, research on the association between an adverse environment from the beginning of life and the subsequent development of non-communicable diseases has allowed an understanding of the origin of some metabolic diseases, such as diabetes and obesity, certain types of cancer, and some disorders in neurodevelopment, educational development, reproductive health, and mental health (13).

Population studies conducted in Brazil estimated a 25.2% rate of obesity (14,15) and a 34.6% rate of overweight among women in 2013 (15). Among pregnant women, birth cohort studies conducted in Pelotas, RS, detected a mean increase of 11.5 kg in pregestational weight between 1982 and 2015 and an increase in the rate of overweight and obesity among women from 22.1 to 47% during the same period (16).

Thus, because of the increase of women with excess weight or obesity and its possible effects on birth weight, the objective of the present study was to assess the association between pregestational maternal body mass index (BMI) and birth weight in a prenatal cohort from Ribeirão Preto, SP, Brazil.

## Material and Methods

This was a prospective study using data from the prenatal cohort of the study “Etiological factors of preterm birth and consequences of perinatal factors for children's health: a birth cohort from two Brazilian cities - BRISA”, conducted in Ribeirão Preto and São Luís (17) in 2010. However, only the Ribeirão Preto data were included and a cross-sectional analysis was performed.

The cohort sample size was calculated according to the reported prevalence of the explanatory variables of the project, which ranged from 10 to 50% and considering a predicted rate of prematurity of 12%. Consequently, 1500 pregnant women were recruited in Ribeirão Preto.

Pregnant women were invited to participate in the study during the first trimester; prenatal visits were held in hospitals and health clinics of the city. Obstetrical ultrasound was performed up to week 20 of gestation to estimate gestational age (GA). During the second trimester, between weeks 20 and 25 GA, they were evaluated using a standardized questionnaire regarding sociodemographic, general health, and reproductive characteristics. On that occasion, a total of 1400 pregnant women reported their pregestational weight, whereas their height was measured.

The participants were reevaluated at childbirth using a second standardized questionnaire when NB anthropometric data were also collected from their medical records. Data were collected from January 2010 to July 2011 for a total of 1370 mother-child pairs. Women whose weight and/or height measurements were not available were excluded from the study, resulting in a sample of 1362 women.

The dependent variable (birth weight) was classified as low when less than 2500 g, adequate when 2500 g and less than 4000 g, and high when 4000 g or more, according to WHO (18).

Pregestational BMI (weight/height^2^), the independent variable, was categorized as malnutrition when below 18.5 kg/m^2^, adequate if 18.5 kg/m^2^ or higher and less than 25 kg/m^2^, overweight if 25 kg/m^2^ or higher and less than 30 kg/m^2^, and obesity if 30 kg/m^2^ or more (19).

**Figure 1:**
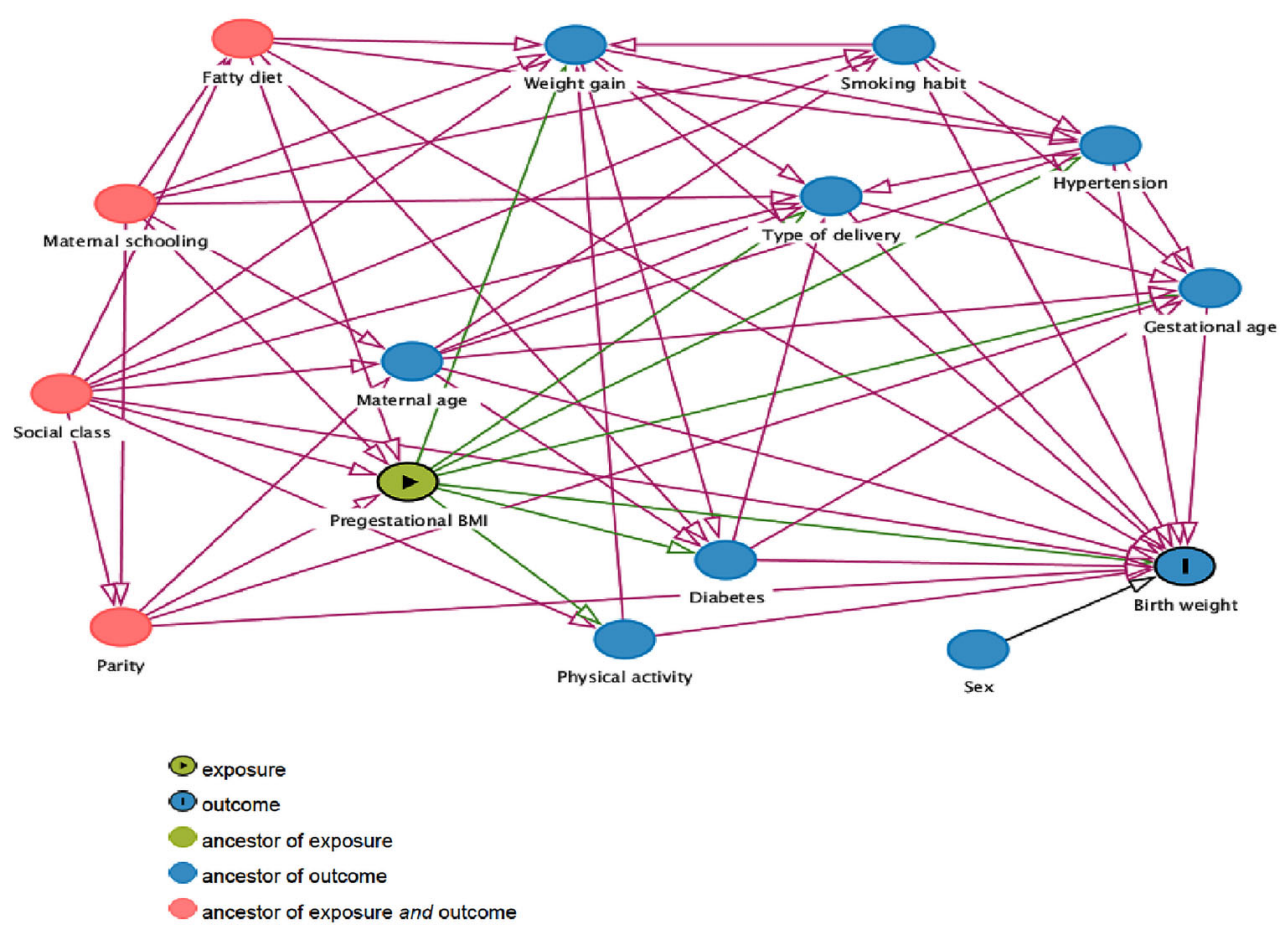
Theoretical model of the association between pre-gestational body mass index (BMI) and birth weight.

To determine the association of pregestational BMI with birth weight, a theoretical model was designed (Figure 1) using Directed Acyclic Graphs (DAG), generated by DAGitty software, version 2.3 (20), which allowed to identify confounding variables to be controlled for.

Maternal variables used in DAG were: schooling (8, 9-11, or ≥12 years of study), age (up to 19 years, 20-34 years, or ≥35 years), economic class based on the instrument elaborated by the Brazilian Association of Research Enterprises (ABEP) (A/B, C, and D/E, A1/A2 being the highest), parity (1, 2-3, and 4 or more children), smoking during pregnancy (at least one cigarette at any point in the pregnancy, yes or no), diet (high or low in fat), level of physical activity during pregnancy (no activity, mild, moderate, or high activity), gestational diabetes (yes or no), gestational hypertension (yes or no), type of delivery (cesarean or vaginal), gestational weight gain (calculated as the difference between weight at the end of pregnancy and weight before pregnancy), gestational age (weeks), and NB sex. The fat content of the diet was assessed using the Block Score (21) and the level of physical activity was assessed using the Short form of the International Physical Activity Questionnaire (IPAQc) (22).

Data are reported as means±SD or proportions, whichever is appropriate. Statistical tests included ANOVA or chi-squared tests. The DAGitty software provided a minimum adjustment model for the estimate of the total effect of the explanatory variable on the outcome, including the following variables: social class, diet, maternal schooling, and parity (Figure 1). Subsequently, a multinomial regression model was used to estimate the crude and adjusted relative risk (RR) of low and high birth weight. All statistical analyses were performed using the statistical package Stata, version 13.0 (StataCorp LP; USA). A P-value <0.05 was considered significant.

The project was approved by the Research Ethics Committee of the University Hospital, Ribeirão Preto Medical School, University of São Paulo (protocol No. 8776/2012), and all subjects gave written informed consent to participate in the study.

## Results

**Figure 2:**
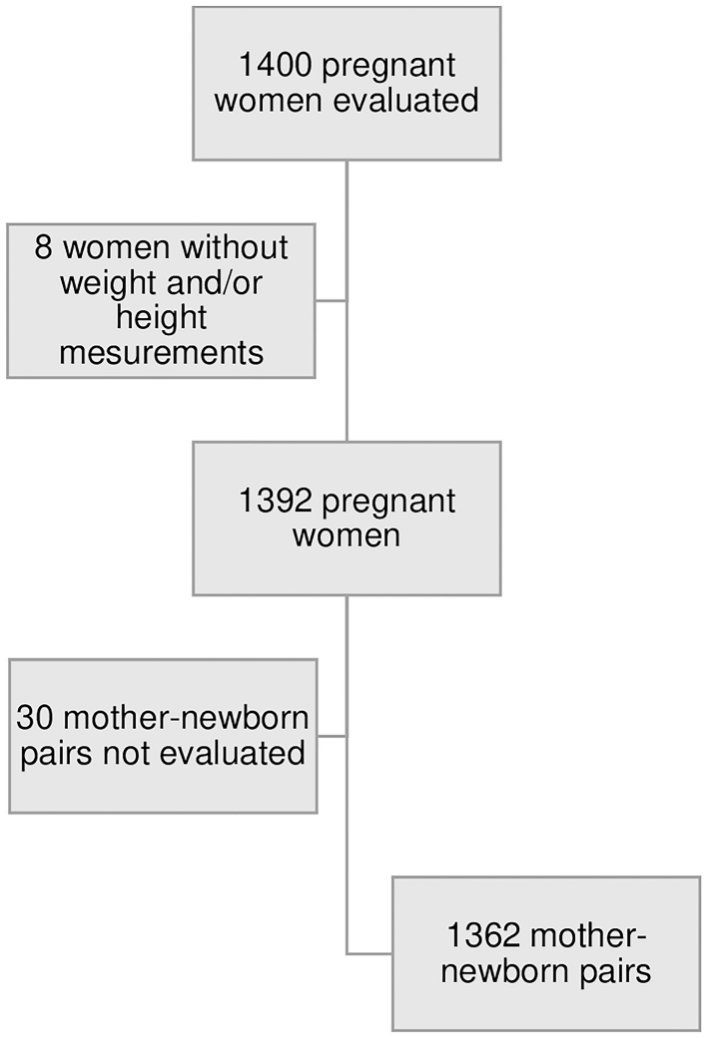
Flowchart of the study population.

Figure 2 presents the flowchart of the study population. Overweight was observed in 25.4% and obesity in 14.2% of pregnant women; 14% were adolescents, 49.3% were primiparous, 8.4% had a lower level of schooling, 28.4% had a diet rich in fat, 12.7% were smokers, 14% had gestational hypertension, 5.3% had gestational diabetes, 16.1% were sedentary, and 59.6% had a cesarean section (Table 1). Mean gestational age at birth was 39 weeks (SD 2.1), the rate of preterm births was 9.7%, and the rate of low birth weight and high birth weight was 7.7 and 5.5%, respectively.

**Table 1:** Maternal and newborn characteristics. BRISA Cohort, Ribeirão Preto, 2010.

Characteristics	n	%
Maternal		
Maternal pregestational BMI^*^		
Malnutrition	101	7.4
Adequate	722	53.0
Overweight	346	25.4
Obesity	193	14.2
Maternal age		
Up to 19 years	192	14.0
20-34 years	1040	75.9
≥35 years	138	10.1
Parity		
1 child	676	49.3
2-3 children	593	43.3
4 or more children	101	7.4
Maternal schooling^*^		
Up to 8 years	114	8.4
9-11 years	856	63.0
≥12 years	388	28.6
Social class (ABEP)		
A/B	365	28.3
C	778	60.2
D/E	149	11.5
Diet (Block score)^*^		
Low in fat	979	71.6
High in fat	389	28.4
Smoking during pregnancy		
Yes	174	12.7
No	1196	87.3
Gestational hypertension		
Yes	192	14.0
No	1178	86.0
Gestational diabetes		
Yes	73	5.3
No	1297	94.7
Level of physical activity^*^		
No activity	217	16.1
Light	426	31.5
Moderate	417	30.8
High	292	21.6
Type of delivery		
Cesarean	817	59.6
Vaginal	553	40.4
Newborns		
Gender		
Female	673	49.1
Male	697	50.9
Weight^*^		
Low	105	7.7
Adequate	1187	86.8
High	75	5.5
Gestational age (weeks, mean ± SD)	39±2.1	

BMI: body mass index; ABEP: Brazilian Association of Research Companies.

*Unknown values were excluded.

Mean maternal BMI and pregestational weight was 24.6 kg/m^2^ (SD 5.3) and 63.7 kg (SD 14.6), respectively, whereas mean weight gain during pregnancy was 14.6 kg (SD 6.1). Mean maternal weight gain during pregnancy decreased with increasing pregestational BMI, being higher than the Institute of Medicine's (IOM) recommendations (23) (Table 2).

**Table 2:** Maternal weight gain during pregnancy according to pregestational maternal BMI. BRISA Cohort, Ribeirão Preto, 2010.

Pregestational maternal BMI (kg/m^2^)	n	Weight gain during pregnancy (kg)	IOM recommendation (kg)
		Mean±SD*	
<18.5	95	16.06±6.10	12.5-18
18.5-24.9	667	15.06±5.77	11.5-16
25-29.9	310	14.19±6.17	7-11
≥30	149	12.29±6.40	5-9.1

BMI: body mass index; SD: standard deviation; IOM: Institute of Medicine. *P<0.001, one-way analysis of variance.

Mean birth weight increased significantly with increasing maternal pregestational BMI (Table 3).

**Table 3:** Birth weight according to maternal pregestational BMI. BRISA Cohort, Ribeirão Preto, 2010.

Pregestational maternal BMI (kg/m^2^)	n	Birth weight (g)
		Mean±SD*
<18.5	101	3066±458*
18.5-24.9	722	3158±524
25-29.9	346	3239±567
≥30	193	3299±636

BMI: body mass index; SD: standard deviation. *P<0.001, one-way analysis of variance.

Maternal pregestational malnutrition was not associated with low birth weight risk, whereas in adjusted models, maternal overweight and obesity were significantly associated with a two-fold increase and a three-fold increase of high weight risks, respectively (Table 4).

**Table 4:** Crude and adjusted relative risks of low weight and high birth weight according to maternal pregestational BMI. Ribeirão Preto, 2010.

Pregestational maternal BMI	Crude Relative Risk		Adjusted Relative Risk^*^	
	Risk for LBWRR (95%CI)	Risk for HBWRR (95%CI)	Risk for LBWRR (95%CI)	Risk for HBWRR (95%CI)
Adequate	1	1	1	1
Malnutrition	1.32 (0.65-2.69)	0.28 (0.04-2.13)	1.70 (0.81-3.55)	0.31 (0.04-2.33)
Overweight	1.14 (0.71-1.83)	2.29 (1.30-4.03)	1.05 (0.63-1.76)	2.13 (1.19-3.80)
Obesity	0.98 (0.52-1.85)	3.43 (1.87-6.29)	1.01 (0.52-1.96)	3.34 (1.80-6.19)

*Adjusted for economic class, maternal schooling, diet, and parity. BMI: body mass index; LBW: low birth weight; HBW: high birth weight; RR: relative risk; CI: confidence interval.

## Discussion

The present findings showed that maternal pregestational overweight and obesity were associated with high birth weight, whereas maternal pregestational malnutrition was not associated with low or high birth weight.

The nutritional transition was responsible for an alarming worldwide increase in overweight and obesity among women of reproductive age (24,25). In the present study, mean pregestational BMI was within the upper limits of normality and almost 40% of the women studied were overweight or obese, in agreement with data reported for other Brazilian cities (16,26–28). These studies show a greater proportion of excess weight than detected in other populations like Peru (29), Holland (11), Lebanon (10), and Indonesia (30), but lower than that detected in the USA (31).

Previous studies have shown an association of increased pregestational BMI with high birth weight (9,11,12,32). In addition, obese women with higher gestational weight gain show increased risks of cesarean delivery, labor induction, and postpartum hemorrhage. On the other hand, macrosomia was associated with a higher rate of admission of newborns to the neonatal intensive care unit and higher perinatal mortality (33).

However, some studies have observed that overweight/obese women also had an increased risk of SGA babies (34), while others showed neither association with low birth weight nor macrosomic babies (35).

The BRISA Cohort study from São Luís-MA (28) estimated that 4 kg/m^2^ gain in pregestational BMI was correlated with a 68 g increase in birth weight. The North American Healthy Start study concluded that for each 1 kg/m^2^ increase in pregestational BMI there was a 5.2 g increase in fat mass, 7.7 g in lean mass, and 0.12% in percent body fat in newborns (31). Soltani et al. (30) detected an adjusted odds ratio of 13.4 for macrosomia in obese Indonesian pregnant women.

In contrast to previous reports (8,9), in the present study, maternal malnutrition was not associated with low or high birth weight. A study of 24,241 pregnancies conducted in Aberdeen, Scotland, reported an adjusted odds ratio of 1.7 (95% CI: 1.2-2.0) for malnourished pregnant women associated with low birth weight (32). Another study of 9,613 births conducted in Argentina detected a weak association between low pregestational BMI and low birth weight by multiple regression models (R^2^ 0.37) (6).

The distribution of birth weight in low, adequate, and high categories was similar to that observed in the São Luis-MA birth cohort in 2010 and in the Pelotas-RS birth cohort in 2015, which strengthens the representativeness of the study (36,37).

Theoretically, since Ribeirão Preto has a high human development index compared with other Brazilian cities (38), the malnourished group is due more to biotype than to calorie deprivation, explaining the lower impact of maternal malnutrition on birth size.

Several strategies have been proposed for the analysis of pregestational maternal weight as an exposure variable to reduce bias, minimize confounding, and quantify the contribution of measurement error. Among these strategies are DAG for selection of variables in multivariable models, the use of a flexible approach for the modeling of pregestational BMI (such as fractional polynomials or restricted cubic splines) to examine U- or J-shaped associations with adverse health outcomes, and the use of quantitative bias analysis to evaluate the potential bias due to measurement error in the self-reported weight. The inclusion of these methods is important because pregestational BMI often has a nonlinear relationship with the outcomes of interest (39).

The main strengths of the present study are its methodology, the use of DAG, and the possibility of comparing its findings with those from other Brazilian cities’ cohorts. A limitation was a potential information bias since pregestational weight was self-reported by the participants. However, a systematic review demonstrated that, even if this type of error occurs, it does not interfere significantly with the results of epidemiological perinatal studies (40). Furthermore, it should be pointed out that the sample size was not originally calculated for BMI assessment and therefore type I error cannot be ruled out.

We conclude that maternal pregestational overweight and obesity were associated with high birth weight in this birth cohort, whereas there was no association between maternal malnutrition and low birth weight. Considering the impact of high birth weight on negative mother-child health outcomes, it is important to reinforce public health policies in order to reduce excess weight among women of reproductive age.
